# Dens Invaginatus in Primary Maxillary Molar: A Rare Case Report and Review of Literature

**DOI:** 10.5005/jp-journals-10005-1152

**Published:** 2012-08-08

**Authors:** Arpana V Bansal, Abhinav Bansal, Vinaya Kumar Kulkarni, Reema Sharma Dhar

**Affiliations:** Reader, Department of Pedodontics and Preventive Dentistry, People's Dental Academy, Bhanpur Bypass Road, Bhopal-462010, Madhya Pradesh, India, e-mail: varpana@yahoo.com; Reader, Department of Periodontics, People's College of Dental Sciences and Research Centre, Bhopal, Madhya Pradesh, India; Associate Professor, Department of Pedodontics and Preventive Dentistry, People's College of Dental Sciences and Research Centre, Bhopal, Madhya Pradesh, India; Reader, Department of Pedodontics and Preventive Dentistry Gurunanak Institute of Dental Sciences and Research, Kolkata, West Bengal, India

**Keywords:** Dens invaginatus, Primary molar, Diagnosis, Anomaly

## Abstract

Dens invaginatus is a rare developmental anomaly. It is unusual to find this anomaly in primary dentition. Diagnosis of this dens invaginatus is important due to possible pulpal involvement. Not only that, simultaneous presence of other dental anomaly may require long-term treatment planning. Dens invaginatus can be detected clinically in the tooth presenting unusual crown morphology or radiographically as radiopacity within tooth. This article describes one of the first case reports of dens invaginatus in primary maxillary second molar in a 5-year-old female patient.

**How to cite this article:** Bansal AV, Bansal A, Kulkarni VK, Dhar RS. Dens Invaginatus in Primary Maxillary Molar: A Rare Case Report and Review of Literature. Int J Clin Pediatr Dent 2012;5(2):139-141.

## INTRODUCTION

Dental anomalies are associated with both the primary and the permanent dentitions and can affect either the morphology or the number of teeth.^1^ Dens invaginatus is one such developmental anomaly. It is also called dens in dente, dilated composed odontoma or gestant odontoma,^2^ resulting from invagination of enamel organ into the dental papilla, beginning at the crown and sometimes extending into the root before calcification occurs.^3,4^

Various explanations have been proposed by different workers for etiology of DI. According to Pindborg,^5^ the etiology of this malformation is unknown; yet the following explanations have been proposed: (i) Delayed focal growth, (ii) stimulation in the area of the tooth bud and (iii) abnormal pressure on tissues surrounding the dental organ. Kronfeld^6^ proposed that DI is caused by a focal failure of growth of the internal enamel epithelium, leading to the proliferation of the surrounding normal epithelium with eventual engulfment of the static area. Oehlers^7^ stated that distortion of the enamel organ occurring during tooth development and results in protrusion of the part of the enamel organ. While some authors believe infection, trauma and genetics to be the reason for DI.^8^

Clinically, dens invaginatus appears in the tooth crown at the site of an anatomical lingual pit susceptible to caries.^9^ Crown of the affected teeth may appear normal in size and shape or may be malformed with a wide apical foremen.^8^ Radiographically, it shows a radiopaque invagination, equal in density to enamel, extending from the cingulum into the root canal. The defects may vary in size and shape from a loop like, pear-shaped or slightly radiolucent structure to a severe form resembling a ‘tooth within a tooth'.^10^ It can be identified easily because infolding of the enamel lining is more radiopaque than the surrounding tooth structure.^3^ Roots of the affected tooth may present smaller dimensions.^11^

Oehlers^7^ described dens in dente according to invagination degree in three forms:

Type 1: An enamel-lined minor form occurs within the crown of the tooth and not extending beyond the cemento- enamel junction.

Type 2: An enamel-lined form which invades the root as a blind sac and may communicate with the dental pulp.

Type 3: A severe form which extends through the root and opens in the apical region without communicating with the pulp.

Dens invaginatus was first observed by De Smit and Demaut^12^ in 1856. Mann^13^ in 1990 described presence of dens invagination in primary molar of a 5-year-old child from fifteen century. Simultaneous presence of other dental anamolies like dentinogenesis imperfecta, germination, taurodontism, microdontia, supernumerary teeth and short roots along with dens invaginatus had been reported in past.^14^ Dens invaginated tooth pose abnormal anatomical configuration. There is presence of thin canals or fissures connecting the invagination to the pulp cavity. This act as pathways for microorganism and irritating agents from oral cavity to seep to pulp leading to alteration in pulp before the development of caries.^11^ Therefore, early diagnosis and management are crucial.

Presence of dens invaginatus in primary dentition is rare.^15^ Only few cases had been reported during computerized search. This article describes one of the first case reports of dens invaginatus in primary maxillary molar.

## CASE REPORT

### History

A 5-year-old female patient reported to the department of pedodontics and preventive dentistry with a chief complain of severe pain and swelling in the left upper back region since 20 days. The patient was having continuous pain and was able to localize the left maxillary second primary molar as the tooth in question. Pain aggravated on chewing food and was relieved on taking medication. The patient's medical history was noncontributory.

## CLINICAL EXAMINATION

**Fig. 1:**
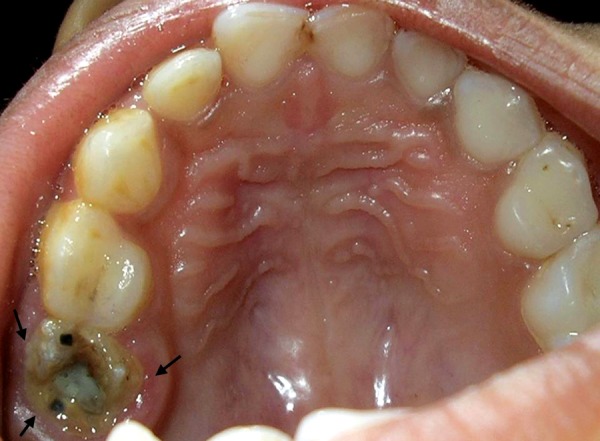
Intraoral view showing grossly carious left primary maxillary second molar (A) arrow indicating intraoral swelling around affected tooth

Extraoral examination revealed diffuse swelling on left side of face. Intraorally, swelling was present on the palatal, buccal and distal side of left primary maxillary second molar (indicated by arrow in Fig:1(A)). The affected tooth was grossly carious (Fig. 1) involving almost all surface of tooth and appeared severely hypoplastic. Exudate was present within the tooth.

## RADIOGRAPHIC EXAMINATION

**Fig. 2:**
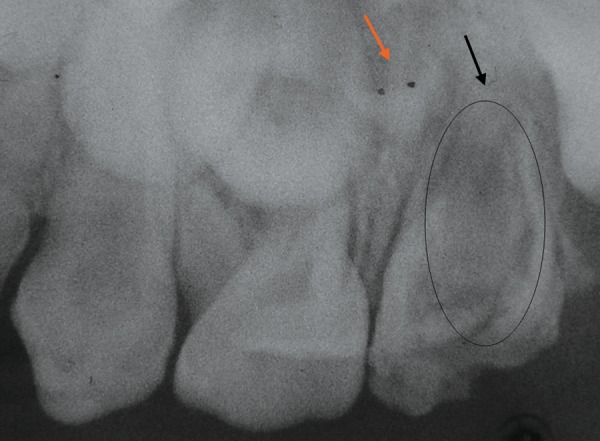
IOPA radiograph showing oval radiopacity within the crown and root portion of primary maxillary second molar (indicated by oval outline) (A) black color arrow indicating open apex of palatal root, (B) orange color arrow indicating rudimentary tooth bud of maxillary second premolar

Intraoral periapical radiograph showed presence of oval shaped radiopacity giving impression of tooth like structure within left primary maxillary second molar (Fig. 2). Thin root canal wall and broad open apex was present with respect to palatal root (indicated by black color arrow in Figure 2A). Mesial and distal roots appear to short. The tooth germ of the maxillary second premolar was smaller in dimensions (rudimentary) than normal, under the root of primary maxillary second molar (indicated by orange color arrow in Figure 2B). Radiographic appearance of the tooth suggested the presence of a dens invaginatus.

## TREATMENT

Considering complex unfavorable root morphology (delicate root canal wall and wide apical foramen) and clinical presentation of case, extraction was considered as treatment of choice. Antibiotic and analgesic was prescribed to alleviate the infection and pain. One week later the tooth was extracted. Unfortunately, owing to badly destruction of tooth surface due to dental caries and thin root wall, it was not possible to remove tooth in one piece. We were able to retrieve tooth only into small coronal and root pieces. An orthodontic treatment plan has been made for space management of the extracted tooth.

## DISCUSSION

Dens invaginatus is a defect in morphological development whereby the coronal enamel and dentin become inverted into the pulp chamber.^16^ In majority of cases dental caries develop inside the dens invaginatus without any clinically detectable lesion. As the enamel lining is thin and is in close proximity to the pulp chamber, a carious lesion easily perforate pulp chamber.^15^ Many treatment regimens have been suggested, such as prophylactically sealing invagination with resin, conventional root canal treatment, combined root canal-surgical treatment, intentional replantation and extraction.^11^

The dental professionals frequently use dental radiographs as an important diagnostic tool. Diagnosis of dens invaginatus in present case was based on radiographic findings. Deep invagination in our case had lead to thin root canal wall and wide open apex. Not only that a lot of destruction of crown structure had occurred due to dental caries. This had made any preventive treatment and root canal treatment impossible in the affected tooth.

The incidence of dens invaginatus ranges from 0.04 to 10% and primarily affects the permanent dentition.^17,18^ In permanent dentition it commonly occurs in maxillary lateral incisors followed by the maxillary central incisors, premolars, canines and less often in the molars.^3^ Invaginations are rare in the mandibular teeth and in primary teeth.^19^ There are only four well-documented cases in primary dentition. One of the earliest case reports (1952) describes dens invaginatus in primary maxillary canine.^20^ Early diagnosis, prompt treatment is vital to prevent any complication caused by invasion of microorganism via thin canals connecting the invagination to the pulp cavity. Initially pulp probably remains uninfected; however, the infection may reach the pulp through the apical opening. Holan^13^ had described dens invaginatus in primary left mandibular canine in 5-year- old child. Owing to pathology in canine, root canal treatment was done. Kupietzky^21^ followed preventive therapy utilizing composite restoration in defected dens invaginated primary maxillary central incisor. Eden et al^16^ reported a case of dens invaginatus in mandibular left second primary molar. Attributable to periapical pathology, the affected molar was extracted. Tooth germ of mandibular second premolar was missing in same patient.

Interestingly, all these cases were in male patient. This is one of the first case reports which describe presence of dens invaginatus in primary maxillary second molar in 5-year-old female patient.

## CONCLUSION

Early diagnosis of dens invagination is of clinical significance. Microorganisms invade the invagination and cause inflammation of the tissue. Inflammatory sequel related to invagination can be avoided by early diagnosis and prophylactic treatment. Radiographs are valuable means of diagnosis along with clinical examination in case of dens invagination.
